# Contrasting Hydraulic Efficiency and Photosynthesis Strategy in Differential Successional Stages of a Subtropical Forest in a Karst Region

**DOI:** 10.3390/plants10122604

**Published:** 2021-11-27

**Authors:** Guilin Wu, Dexiang Chen, Zhang Zhou

**Affiliations:** Hainan Jianfengling Forest Ecosystem National Field Science Observation and Research Station, Research Institute of Tropical Forestry, Chinese Academy of Forestry, Guangzhou 510520, China; wuguilin15@mails.ucas.ac.cn (G.W.); dchen@caf.ac.cn (D.C.)

**Keywords:** forest succession, hydraulic conductance, photosynthetic rate, leaf turgor loss point, wood density, trade-off

## Abstract

Understanding the successional process from a disturbed forest to a mature forest is essential for species recovery and conservation initiatives. The resource acquisition and drought tolerance of plants can be instructive to predictions of species abundance and distribution for different forests. However, they have not been adequately tested at different successional stages in karst regions. Here, we selected seven dominant species in an early-succession forest and 17 species in a late-succession forest in a karst region of southwestern China. Resource acquisition-related traits such as hydraulic conductivity and photosynthetic rate, and drought tolerance-related traits, including turgor loss point and wood density, were measured. We found that species in the early-succession forest had a higher hydraulic conductance and photosynthetic rate than those in the late-succession forest, while leaf water potential at turgor loss point and wood density showed nonsignificant differences between the two forests. In addition, we observed a significant negative relationship between photosynthetic rate and drought tolerance in the early-succession forest, which was not identified in late-succession forests. Our study indicates that resource acquisition rather than drought tolerance was the key factor explaining plant distributions in forests at different successional stages in karst regions. We also suggest that the resource acquisition and drought tolerance trade-off hypothesis is not always supported for karst region species. Our study could inform about the design of species replacements in successional forests and provide forest management and restoration guidelines for karst regions.

## 1. Introduction

Southwest China contains one of the world’s largest continuous karst zones, with a total area more than 500,000 km^2^. The forests in this karst zone are extremely species-rich and host many endemic plant species [1]. Most primary forests in karst regions have been substantially disturbed by human activities, and substantial forest degradation has occurred in southwest China [2,3,4]. Therefore, understanding the successional process from a disturbed forest to a mature forest is fundamental for species recovery and conservation. However, the pivotal mechanisms of these plant groups at different successional stages have not been well-studied in karst regions. 

The functional traits of plants can represent the capacity for resource acquisition and drought tolerance, which are key characteristics used to explain the adaptation of plant groups to different environments [5,6]. Especially, stem hydraulic conductance and photosynthetic rate represent water transport efficiency and carbon fixation capacity in plants, respectively, indicating the resource acquisition ability of species. Fast-growing species usually have higher hydraulic conductance and photosynthetic rate, indicating a higher resource acquisition ability [6,7,8]. For example, species in an open forest where light is not limited would have a higher photosynthetic rate and hydraulic conductance than understory species, leading to a higher growth rate for species in an open forest [8,9]. The leaf water potential at turgor loss point (*ψ_tlp_*) and wood density (*WD*) are increasingly being used as functional traits for determining drought tolerance [10,11,12,13]. Species from drier sites typically have lower leaf water potentials at turgor loss point and wood density [14,15,16,17]. 

Many studies have suggested that the functional traits underlying the resource acquisition and drought tolerance trade-off of plants might be instructive for predictions of species abundance and distribution along a successional forest [6,8,18,19,20]. In general, early-succession forests are formed by shrubs and grasses, which usually have higher photosynthetically active radiation than late-succession forests. Therefore, species at early-succession forests should grow faster to outcompete their neighbors at the cost of drought tolerance [5]. Species in late-succession forests try to maintain their niche with high drought tolerance, which increases the survival of these plants but leads to a lower carbon fixation rate [6]. Therefore, species in the early-succession stage usually have a higher photosynthetic rate associated with lower drought tolerance than species in the late-succession stage [21,22]. This trade-off has also been reported in many other studies [23,24,25].

Unlike environments in previous studies, karst regions are formed by the dissolution of limestone and other soft rocks. The soil in these areas is generally shallow, and rapid subterranean drainage results in water loss; thus, the species inhabiting karst regions are frequently at risk of drought [26,27]. These species need to have high water transport efficiency and carbon accumulation when water is available (e.g., precipitation) while maintaining high drought tolerance at other times (e.g., shortly after precipitation). Therefore, we expect that species in karst regions have a high photosynthetic capacity associated with a high drought tolerance, suggesting a non-trade-off between resource acquisition and drought tolerance. A few studies tested this relationship, and their results varied. Previous studies found a trade-off between hydraulic efficiency and drought tolerance in karst regions [28,29], while another study did not find this relationship across 17 species within different topography [30]. Hence, investigating the resource acquisition and drought tolerance and evaluating the relationship between them at different successional stages may allow for a more comprehensive understanding of plant distribution patterns in karst regions.

Here, we selected 7 dominant species in an early-succession forest and 17 dominant species in a late-succession forest in a karst region. We measured stem hydraulic conductance, photosynthetic rate, leaf water potential at turgor loss point, wood density and specific leaf area for all species. Specifically, we aimed to test the following hypotheses: (1) Species in the early-succession forest are fast-growing and have a lower drought tolerance than species in the late-succession forest. (2) There is no trade-off between photosynthetic rate and drought tolerance in karst regions (neither in early- nor in late-succession forests). 

## 2. Materials and Methods 

### 2.1. Study Site and Species

**Figure 1:**
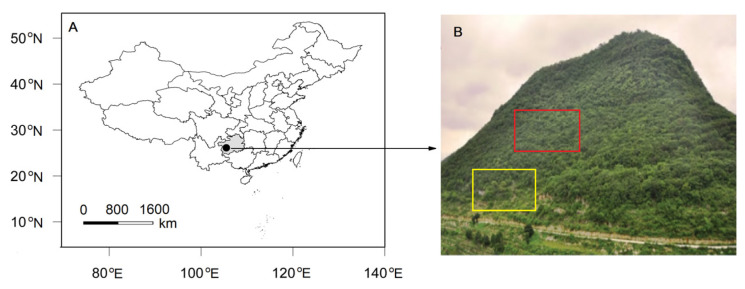
Location of Tianlong Mountain (**A**) and early (yellow square frame) and late (red square frame)-succession communities (**B**) at this mountain.

The study was carried out at Tianlong Mountain (105°45′50″E, 26°14′40″N, 1, 402-1, 512 m in altitude) in Puding County (Figure 1A). Tianlong Mountain is located in central Guizhou Plateau in southwestern China. According to records from the Puding meteorological station from 1961 to 2008, this area is characterized by a typical subtropical monsoon climate, with a mean annual temperature of 15.1 °C, ranging from 5.4 °C in the coldest month (January) to 22.9 °C in the warmest month (July). The mean annual precipitation is 1367 mm, of which more than 70% occurs from May to September. Secondary evergreen and deciduous broad-leaved mixed forests are distributed in the middle and top of Tianlong Mountain, where there are fewer human disturbances. Degraded shrubs, tussocks and grasslands occur at the middle and foot of the mountain [31,32]. The plots (2 ha, 200 m horizontal × 100 m vertical) for both early- and late-succession forests are on the southern aspect and at a 31° slope (Figure 1B). Limestone outcrops are widely distributed, thereby resulting in a soil coverage of 55%. The soil is a brown limestone soil with a shallow soil depth of less than 50 cm [33]. In our study, we selected 24 dominant species, including 7 in early- and 17 (Table 1) in late-succession forests based on the previous studies in the two plots [31,32]. To avoid the impact of seasonal drought on the ecophysiological traits of these species, all measurements were conducted from 7 July to 29 August. 

### 2.2. Gas Exchange Measurement

Leaf gas exchange was measured between 9:00 and 11:00 using a portable photosynthesis system equipped with a CO_2_ injector (Li6400, Li-Cor, Lincoln, NE, USA), on clear days in August. Three to five individuals were selected for each species, and five sun-exposed mature leaves were selected from each individual for photosynthetic measurements. For shrubs, we conducted gas exchange measurements on the plants without cuts. For top trees, we first cut the sun-exposed branches with approximately 1 cm in diameter, placed the stem immediately in water and then quickly measured the gas exchange. Based on preliminary trials, the photosynthetic photon flux density was set at 1500 mol m^−2^ s^−1^ to ensure that light-saturated photosynthesis rates were reached for the study’s species. Ambient CO_2_ was maintained at 400 mol mol^−1^, and leaf temperature was maintained at 25 °C for all measurements. To avoid the impact of vapor pressure deficit on gas exchange, we also controlled the relative humidity in the chamber from 60% to 70%. Before the data were recorded, the leaves were exposed to the above conditions for approximately 5–10 min to allow the photosynthetic parameters to stabilize. Some species had small leaves (e.g., leaves from *Myrsine africanac* and *Pyracantha fortuneana*); therefore, leaf area correction was performed after the measurements. Leaf area was scanned on a flatbed scanner (Scanjet 4500c; HP, Berkshire, UK) and then determined with ImageJ software (version 1.41, Abramoff, 2004).

### 2.3. Stem Hydraulic Conductivity, Sapwood Density, Specific Leaf Area, and Leaf Area and Sapwood Area Ratio

Ten terminal branches (8–10 mm in diameter, 1–2 years old) were sampled early in the morning from three to five mature individuals for each species, sealed in black plastic bags with a moist towel and immediately transported to the laboratory. All of the stem samples were recut under water, and the cut ends were trimmed with a razor blade. The length of stem segments for the measurement of stem hydraulic conductivity was 30–40 cm.

Stem hydraulic conductivity was measured using the method described by previous study [34]. In an airconditioned laboratory (26 °C), branch segments were flushed at a pressure of 0.1 MPa for 20 min to remove air embolisms. Hydraulic conductivity per unit pressure gradient (*K_h_*) is equal to the ratio between water flux through an excised stem segment and the pressure gradient causing the flow. Sapwood specific conductivity (*K_S_*, kg m^−1^ s^−1^ MPa^−1^) is equal to *K_h_* divided by the sapwood cross-sectional area. Leaf-specific hydraulic conductivity *(K_L_*, kg m^−1^ s^−1^ MPa^−1^) was calculated as the ratio of *K_h_* to the leaf area, which is a measure of the hydraulic sufficiency of the stem to supply water to distal leaves. Wood density (*WD*) was measured after removing the bark or pith of these branches; the fresh sapwood was then immersed in distilled water overnight, allowing the sample to saturate. The volume of sapwood was determined by water displacement, and the dry mass was determined after oven-drying at 65 °C for 72 h. Wood density was the ratio of dry mass to fresh volume. Leaf area was scanned on a flatbed scanner (Scanjet 4500c; HP, Berkshire, UK) and then determined with ImageJ software (version 1.41, Abramoff, 2004). The leaf area to sapwood area ratio (*A_L_*/*A_S_*; m^2^ cm^−2^) was calculated as the ratio of leaf area attached per unit of sapwood cross-sectional area.

### 2.4. Pressure–Volume Relations

Terminal branches were harvested from three to five individuals for each species. Branch ends were recut underwater and rehydrated until leaf water potential exceeded −0.05 MPa, after which leaves were detached for pressure–volume curve determination. Leaves were first weighted to obtain the initial fresh mass and then immediately placed in a pressure chamber (PMS, Corvallis, OR, USA). Leaf weight and water potential were measured periodically during the slow desiccation of the sample in the laboratory. After all of the balanced pressure–weight measurements, the leaves were oven-dried for 72 h at 65 °C to determine the dry weight. Leaf water potential at turgor loss point (*ψ_tlp_*) was determined by a pressure–volume relationship analysis program developed by [35].

### 2.5. Statistical Analyses

Differences in photosynthetic traits and drought tolerance between the two successional groups were tested with a one-way ANOVA. The relationships between photosynthetic rate and drought tolerance traits were analyzed using a linear regression analysis. Multivariate associations of the eight functional traits (*A_n_*, *g_s_*, *K_S_*, *K_L_*, *A_L_/A_S_*, *ψ_tlp_*, *WD* and *SLA*) for the 24 species were analyzed with a principal component analysis (PCA). R studio version 4.0.3 [36] was used for all analyses and graphs.

## 3. Results

### 3.1. Resource Acquisition and Drought Tolerance Traits in a Forest at Different Successional Stages

**Figure 2:**
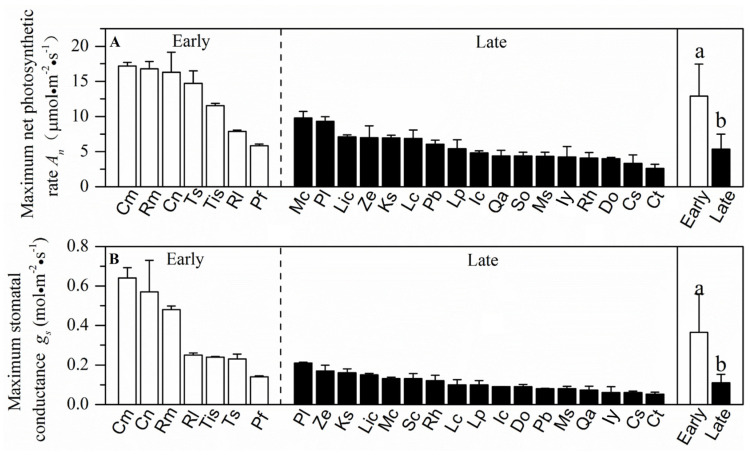
Mean values (±SE) of maximum net photosynthetic rate (*A_n_*) and maximum stomatal conductance (*g_s_*) for 24 wood species in two successional communities. White and black bars represent species from the early- and late-succession stages, respectively. Different letters at the top of each column denote significant differences among communities (*p* < 0.05).

**Figure 3:**
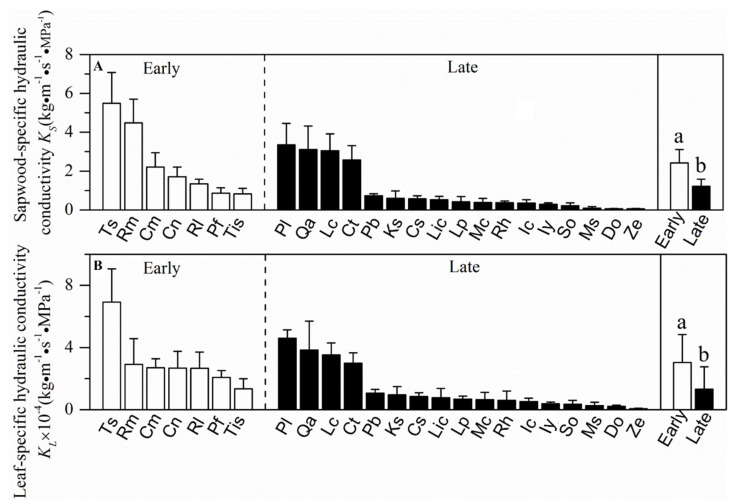
Mean values (±SE) of sapwood-specific hydraulic conductance (*K_S_*) and leaf-specific hydraulic conductance (*K_L_*) for 24 wood species in two successional communities. White and black bars represent species from the early- and late-succession stages, respectively. Different letters at the top of each column denote significant differences among communities (*p* < 0.05).

**Figure 4:**
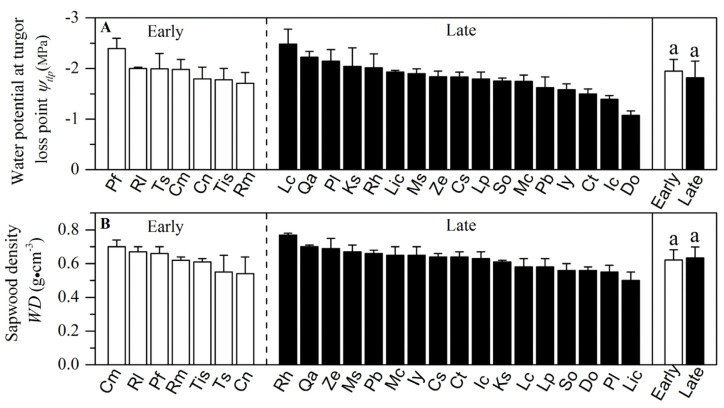
Mean values (±SE) of water potential at turgor loss point (*ψ_tlp_*) and wood density (*WD*) for 24 wood species in two successional communities. White and black bars represent species from the early- and late-succession stages, respectively. Different letters at the top of each column denote significant differences among communities (*p* < 0.05).

**Figure 5:**
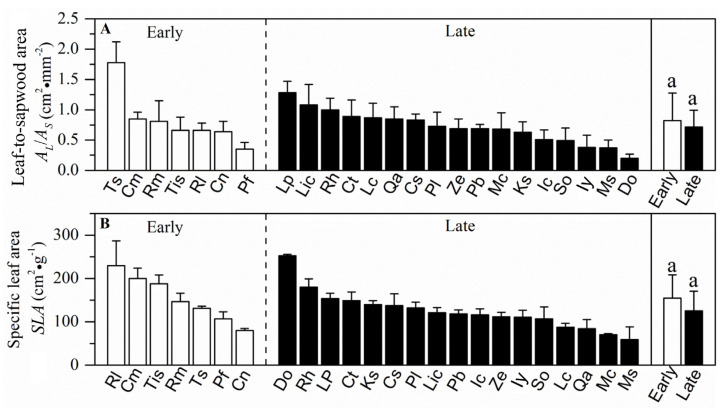
Mean values (±SE) of leaf-to-sapwood area (*A_L_/A_S_*) and specific leaf area (*SLA*) for 24 wood species in two successional communities. White and black bars represent species from the early- and late-succession stages, respectively. Different letters at the top of each column denote significant differences among communities (*p* < 0.05).

Sapwood-specific conductivity, leaf-specific conductivity, photosynthetic rate and stomatal conductance in the early-succession forest were significantly higher than plants grown in the late-succession forest (*p* < 0.05, Figure 2 and Figure 3). In contrast, wood density, leaf water potential at turgor loss point, leaf-to-sapwood area and specific leaf area showed nonsignificant differences between the two successional communities (*p* > 0.05, Figure 4 and Figure 5).

**Figure 6:**
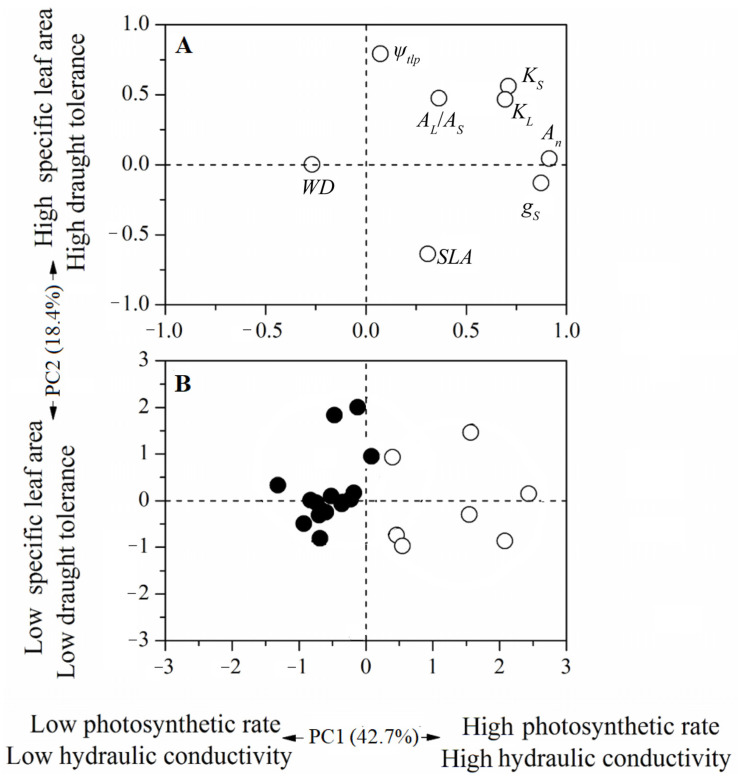
Principal component analyses (PCA) for (**A**) 8 traits and (**B**) 24 woody species for the two axes. White and black circles indicate species from the early- and late-succession stages, respectively, in the graph below (**B**).

A principal component analysis (PCA) was employed to evaluate how the traits of hydraulics, photosynthesis and drought tolerance were associated for subtropical karst species at different successional stages. PC1 explained 42.7% of the variation in traits, showed strong positive loadings for plant hydraulic (*K_S_* and *K_L_*) and photosynthetic traits (*A*_n_ and *g*_s_), and had a negative loading of wood density. Leaf turgor loss point was positively loaded for PC2 (explained 18.4% of the variation in traits) and negatively loaded by specific leaf area. Plant species from different successional stages were well-separated along PC1. In contrast to species in the late-succession forest, early-succession species showed higher hydraulic conductivity, photosynthetic rate and stomatal conductance (Figure 6).

### 3.2. Relationship between Photosynthetic Rate and Drought Tolerance at Different Successional Stages

**Figure 7:**
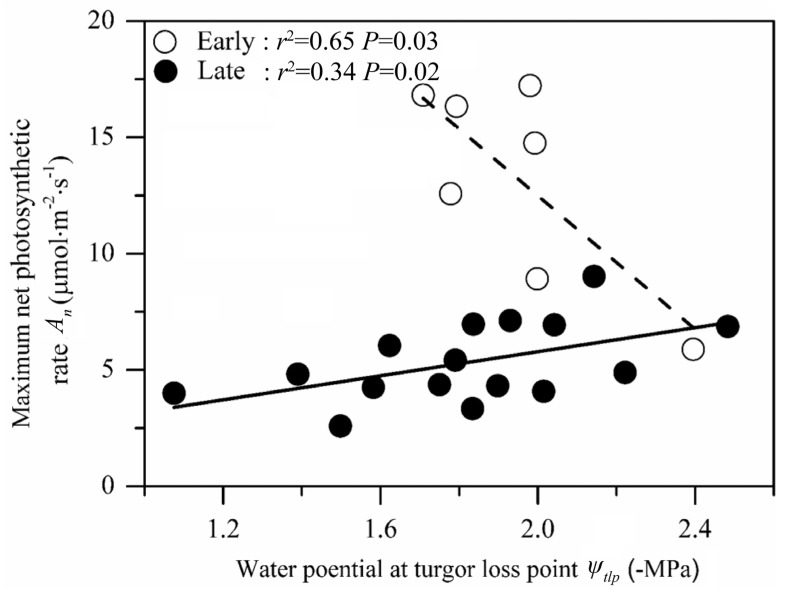
Relationship between maximum net photosynthetic rate (*A_n_*) and water potential at turgor loss point (*ψ_tlp_*). The white circles and the dashed line represent species in the early-successional stage, while the black circles and the solid line represent species in the late-successional stage. Both show a significant relationship (*p* < 0.05).

We found nonsignificant relationships between photosynthetic and drought tolerance traits for all species (*p* > 0.05, Figure 7), including species in both early- and late-succession forests. We showed that photosynthetic rate (*A_n_*, growth trait) and leaf water potential at turgor loss point (*ψ_tlp_*, drought tolerance trait) were negatively correlated in the early-succession forest (*p* < 0.05, Figure 7). In contrast, photosynthetic rate showed a significantly positive relationship with the absolute value of leaf water potential at turgor loss point (drought tolerance) for species in the late-succession forest (*p* < 0.05, Figure 7). 

## 4. Discussion

We found that species in the early-succession forest showed a higher hydraulic conductivity and carbon fixation capacity than species in the late-succession forest, but the species in both forest types showed similar drought tolerance. Thus, the first hypothesis that species in the early-succession forest are fast-growing and have lower drought tolerance than species in the late-succession forest (H1) was partly supported. We found a negative relationship between the photosynthetic and drought tolerance traits for species in the early-succession forest. In contrast, we observed a positive relationship between the photosynthetic rate (*A_n_*) and the absolute value of leaf water potential at turgor loss point (*ψ_tlp_*) in the late-succession forest. Therefore, the hypothesis that species in both succession forests do not show a trade-off between photosynthetic rate and drought tolerance in karst regions (H2) was partially supported. Overall, our study revealed that resource acquisition (*A_n_*, *g_s_*, *K_S_* and *K_L_*) rather than drought tolerance (*ψ_tlp_*, *WD*) was the key factor in explaining plant distributions at different successional stages in karst regions. Our results also suggested that a trade-off between resource acquisition and drought tolerance was not always observed in karst regions. These findings improve our understanding of species replacement in successional forests and provide guidelines for forest management and rebuilding in karst regions. 

### 4.1. Resource Acquisition and Drought Tolerance Traits at Different Successional Stages

When compared to species in late-succession forests, plants distributed at early-successional stages have a higher stem hydraulic conductance and photosynthetic rate. Our results were consistent with previous studies [6,37]. Disturbed by human activity, primary forests are destroyed and open sites are created, resulting in the establishment of fast-growing and light-demanding species, such as *Toona sinensis* and *Coriaria nepalensis*. Higher stem hydraulic conductance enables plants to transport water more effectively to the stomata, thus enabling stomata to remain open to let more CO_2_ in, leading to a higher photosynthetic rate [38,39,40]. These higher resource acquisition capacities in early-succession forests enable species to quickly occupy these open sites, while late-successional species might invest more in survival rather than growth with limited resources [5,41], leading to lower *A_n_*, *g_s_*, *K_S_* and *K_L_*. However, we did not observe higher wood density for species in late-succession forests. Perhaps other characteristics related to survival strategies were critical for species in late-succession forests, such as higher pit area or diameter [42]. This deserves future study in karst regions. Unexpectedly, higher hydraulic conductivity in the early-succession forest did not lead to higher *A_L_*/*A_S_*, which was inconsistent with previous study [6]. A potential explanation for this is that a higher leaf area supported by certain sapwood would increase the risk of dieback.

Leaf water potential at turgor loss point indicates the leaf-level desiccation tolerance of plants from different rainfall sites [14]. This trait was particularly used to describe drought adaptions in plants [43]. In our study, we did not observe a significant difference in leaf water potential at turgor loss point between the two successional forests. This might occur because of similar SLA for species in both successional forests. As SLA suggests the dry matter content degree [44], higher dry matter content enables plants to maintain living tissues under drought risk (low *ψ_tlp_*, [45]). 

A PCA analysis further strengthened the notion that plant hydraulic conductivity and photosynthetic traits (PC1), rather than turgor loss point (PC2), might be the key factors underlying species replacement along karst forest succession (Figure 6). Along PC1, early-succession species exhibited typical fast-growing characteristics, such as higher leaf- and sapwood-specific conductance and photosynthetic rate than species in the late-succession forest. 

### 4.2. Relationship between Photosynthetic Rate and Drought Tolerance at Different Successional Stages

The resource acquisition and drought tolerance trade-off hypothesis suggests that species with a higher carbon fixing capacity would have lower drought tolerance [22,46]. This trade-off has been observed in previous studies, for example, the trade-off in xylem between the efficiency to transport water and the ability to resist embolism (drought tolerance) [6,29,47,48], between photosynthetic rate and the ability to resist embolism [49], and between hydraulic safety efficiency and the growth of tissues [50]. However, inconsistent with these previous studies, we even found a positive relationship between photosynthetic rate and leaf water potential at turgor loss point in the late-succession forests, indicating that species had a higher carbon fixation ability associated with a high drought tolerance. The potential explanation for this non-trade-off relationship could be as follows: In the karst regions in our study, mean precipitation was not low (e.g., annual precipitation was 1367 mm). However, the soils in karst areas are generally shallow, and rapid subterranean drainage results in water loss; hence, the plants experience drought risk shortly after precipitation. Species in karst regions must have high photosynthetic capacity when water is available (e.g., precipitation) and high drought tolerance when at risk of drought (e.g., a short period after precipitation). This would result in a non-trade-off between photosynthetic rate and drought tolerance for species in karst regions or even a significant positive relationship between resource acquisition and drought tolerance traits at the late-succession stage. Similar results were also observed by previous study [51] who found combined high leaf hydraulic efficiency (resource acquisition) and safety (drought tolerance) in *Caragana* species adapted to a low mean annual precipitation. They suggested that these species should have a high photosynthetic rate during the short water availability period during spring to produce flowers and mature seeds, leading to high leaf hydraulic efficiency. To survive in severely dry summers, they also need to have a high drought tolerance. Furthermore, in a recently published paper [52], which explored the relationship between xylem hydraulic efficiency and drought tolerance on a global scale. They found that climatic seasonality was critical in weakening such trade-offs. For example, they found that seasonal drought and seasonal precipitation may favor species with optimized xylem to confer both drought resistance to survive the dry season and an efficient water transport and high photosynthetic rate to support fast growth during the wet season. This also might be the case for the non-trade-off in our study. 

## 5. Conclusions

In this study, we found that resource acquisition rather than drought tolerance was the key factor explaining plant distributions along forest succession in karst regions. In addition, this study demonstrated that a trade-off between resource acquisition and drought tolerance might not always be supported in karst region species. Our study could inform about species replacement initiatives in successional forests and provide forest management and restoration guidelines for karst regions. 

## Figures and Tables

**Table 1:** Characteristics of 24 woody species tested in the present study.

Species	Family	Code	Sampling Site	Successional Stage
*Campylotropis macrocarpa*	Fabaceae	Cm	Open site	Early
*Coriaria nepalensis*	Coriariaceae	Cn	Open site	Early
*Tirpitzia sinensis*	Linaceae	Tis	Open site	Early
*Rhamnus leptophylla*	Rhamnaceae	Rl	Forest margins	Early
*Pyracantha fortuneana*	Rosaceae	Pf	Open site	Early
*Rhamnella martini*	Rhamnaceae	Rm	Open site	Early
*Toona sinensis*	Meliaceae	Tos	Open site	Early
*Pittosporum brevicalyx*	Pittosporaceae	Pb	Understory	Late
*Ilex coralline*	Aquifoliaceae	Ic	Understory	Late
*Stachyurus obovatus*	Stachyuraceae	So	Understory	Late
*Myrsine semiserrata*	Primulaceae	Ms	Understory	Late
*Itea yunnanensis*	Iteaceae	Iy	Understory	Late
*Rhamnus heterophylla*	Rhamnaceae	Rh	Understory	Late
*Daphne odora*	Thymelaeaceae	Do	Understory	Late
*Machilus cavaleriei*	Lauraceae	Mc	Understory	Late
*Kalopanax septemlobus*	Araliaceae	Ks	Forest canopy	Late
*Lithocarpus confinis*	Fagaceae	Lic	Forest canopy	Late
*Lindera pulcherrima*	Lauraceae	Lp	Forest canopy	Late
*Celtis sinensis*	Cannabaceae	Cs	Forest canopy	Late
*Carpinus turczaninowii*	Betulaceae	Ct	Forest canopy	Late
*Platycarya longipea*	Juglandaceae	Pl	Forest canopy	Late
*Quercus acutissima*	Fagaceae	Qa	Forest canopy	Late
*Lindera communis*	Lauraceae	Lc	Forest canopy	Late
*Zanthoxylum esquirolii*	Rutaceae	Ze	Forest canopy	Late

## Data Availability

The data presented in this study are openly available.
